# From Diarrhea to Bacteremia: Extended-Spectrum Beta-Lactamase (ESBL)-Producing Shigella in a Rare Clinical Scenario

**DOI:** 10.7759/cureus.78566

**Published:** 2025-02-05

**Authors:** Usamah Al-Anbagi, Muna Al Maslamani, Mohamed Aboukamar, Manal Hamed, Emad Elmagboul, Riyas Kayanattath, Abdulqadir J Nashwan, Aram Salehi

**Affiliations:** 1 Internal Medicine, Hamad Medical Corporation, Doha, QAT; 2 Infectious Diseases, Hamad Medical Corporation, Doha, QAT; 3 Pathology and Laboratory Medicine, Hamad Medical Corporation, Doha, QAT; 4 Microbiology, Hamad Medical Corporation, Doha, QAT; 5 Nursing and Midwifery Research, Hamad Medical Corporation, Doha, QAT

**Keywords:** blood culture, ertapenem, extended spectrum beta-lactamase (esbl), shigella sonnei (s. sonnei), shigellemia, stool culture

## Abstract

Though globally prevalent, *Shigella *infections rarely progress to bacteremia (shigellemia), particularly in immunocompetent individuals. Here, we report a case of shigellemia with extended-spectrum beta-lactamase (ESBL) in Qatar, involving a 53-year-old immunocompetent male with no significant medical history. The patient presented with a one-day history of frequent loose stools, fever, and mild central abdominal pain. Stool and blood cultures confirmed *Shigella sonnei*infection and shigellemia with ESBL production. The patient tested negative for human immunodeficiency virus (HIV), had normal immunoglobulin levels, and was successfully treated with a 10-day course of ertapenem, achieving full recovery. This case underscores the importance of considering shigellemia in patients with severe gastrointestinal symptoms, even without immunosuppression. It highlights the need for prompt diagnosis, antibiotic susceptibility testing, and targeted antimicrobial therapy, especially given the rising prevalence of antibiotic-resistant strains. A thorough investigation of predisposing factors and patient education on hygiene practices remains essential to prevent transmission and mitigate community outbreaks.

## Introduction

*Shigella *infections represent a significant global health concern, responsible for approximately 188 million cases and 164,000 deaths annually [1]. They are a leading cause of invasive diarrhea in children, particularly in resource-limited settings [2]. Among the four *Shigella *species (*Shigella dysenteriae*, *Shigella flexneri*, *Shigella boydii*, and *Shigella sonnei*), *Shigella flexneri* predominates in low-income regions, while *S. sonnei* is increasingly prevalent in areas undergoing economic transition [3].

Transmission occurs primarily via the fecal-oral route, through household or sexual contact, and ingesting contaminated food or water [4]. Although *Shigella* typically causes gastrointestinal symptoms, bacteremia is a rare complication, with an incidence of 0-7% [5,6]. This complication is more common in vulnerable populations, such as young children, older adults, and individuals with comorbidities, including human immunodeficiency virus (HIV) and other immunocompromising conditions [6,7]. The emergence of antibiotic-resistant *Shigella *strains, driven by the horizontal transfer of resistance genes, presents a growing therapeutic challenge, especially with resistance to fluoroquinolones, cephalosporins, and azithromycin [8,9].

This report highlights a case in Qatar of *Shigella sonnei*-induced bacteremia with extended-spectrum beta-lactamase (ESBL) production in an immunocompetent adult. It underscores the importance of considering shigellemia in severe gastrointestinal presentations, even in the absence of traditional risk factors. Moreover, it emphasizes the critical need for prompt diagnosis, antibiotic susceptibility testing, and targeted treatment to address the rising prevalence of antibiotic resistance.

## Case presentation

A 53-year-old previously healthy male with no significant comorbidities, apart from well-controlled hypertension (managed with amlodipine 5 mg), presented to the emergency room and reported experiencing loose stools for one day. The patient described his stools as profuse, frequent, yellowish, and without blood or mucus. He also reported a one-day history of fever associated with mild central abdominal pain. He denied vomiting, extra-intestinal symptoms, or any previous similar episodes. He had no history of recent travel, sick contacts, or extra-marital relationships, and his family history was non-contributory.

On examination, the patient was febrile (38.9°C), slightly tachypneic (18 breaths/min), with normal oxygen saturation (98% on room air) and a blood pressure of 102/65 mmHg. Other clinical examinations were unremarkable. Given the patient’s condition, a decision was made to admit him for further management.

Initial laboratory investigations revealed mild leukocytosis (WBC 10.2 × 10⁹/L), mild acute kidney injury (creatinine 187 µmol/L, urea 11.7 mmol/L), hyponatremia (sodium 128 mmol/L), elevated lactate (4 mmol/L) likely due to sepsis, and high C-reactive protein (CRP) (283 mg/L) (Table 1). The chest X-ray (CXR) and electrocardiogram (ECG) showed no significant abnormalities.

**Table 1:** Laboratory investigations Hb: hemoglobin; PLT: platelet; CRP: C-reactive protein; PCT: procalcitonin; K: serum potassium; Na: serum sodium; urea: serum urea; SCr: serum creatinine; HbA1c: hemoglobin A1c; Alb: serum albumin; TP: serum total protein; AST: aspartate aminotransferase; ALT: alanine aminotransferase; ALP: alkaline phosphatase; TBil: serum total bilirubin; Cl: serum chloride; HCO₃: serum bicarbonate; HIV: human immunodeficiency virus

Parameters	On admission	Third day	On discharge	Reference values
Total leukocytes	10.2	5.2	7	(6.2 x10^3/uL)
Hemoglobin (gm/dL)	16.3	13.8	13.8	(13-17 gm/dL)
Platelet (x10^3/uL)	221	208	428	(150-410 x10^3/uL)
CRP (mg/L)	283	139	5	(0-5 mg/L)
Procalcitonin (ng/mL)	7.68	-	0.13	(<0.5 ng/mL)
Serum potassium K (mmol/L)	3.8	3.6	4	(3.5-5.3)
Serum sodium (mmol/L)	128	137	138	(133-146)
Serum urea (mmol/L)	11.7	2.5	3.2	(2.5-7.8)
Serum creatinine (umol/L)	187	69	59	(62-106)
HbA1c	5.5	-	-	<6%
Serum albumin (gm/L)	33		33	(35-50)
Serum total protein (gm/L)	75	-	77	(60-80)
AST (IU/L)	35	-	146	(0-41)
ALT (IU/L)	28	-	91	(0-41)
Alkaline phosphatase (U/L)	70	-	118	(40–129)
Serum total bilirubin (mg/dl)	10	-	4	(0-21)
Serum chloride (mmol/L)	94	109	105	(95-108)
Serum bicarbonate (mmol/L)	21	21	26	(22-29)
HIV	Negative	-	-	Negative

**Figure 1:**
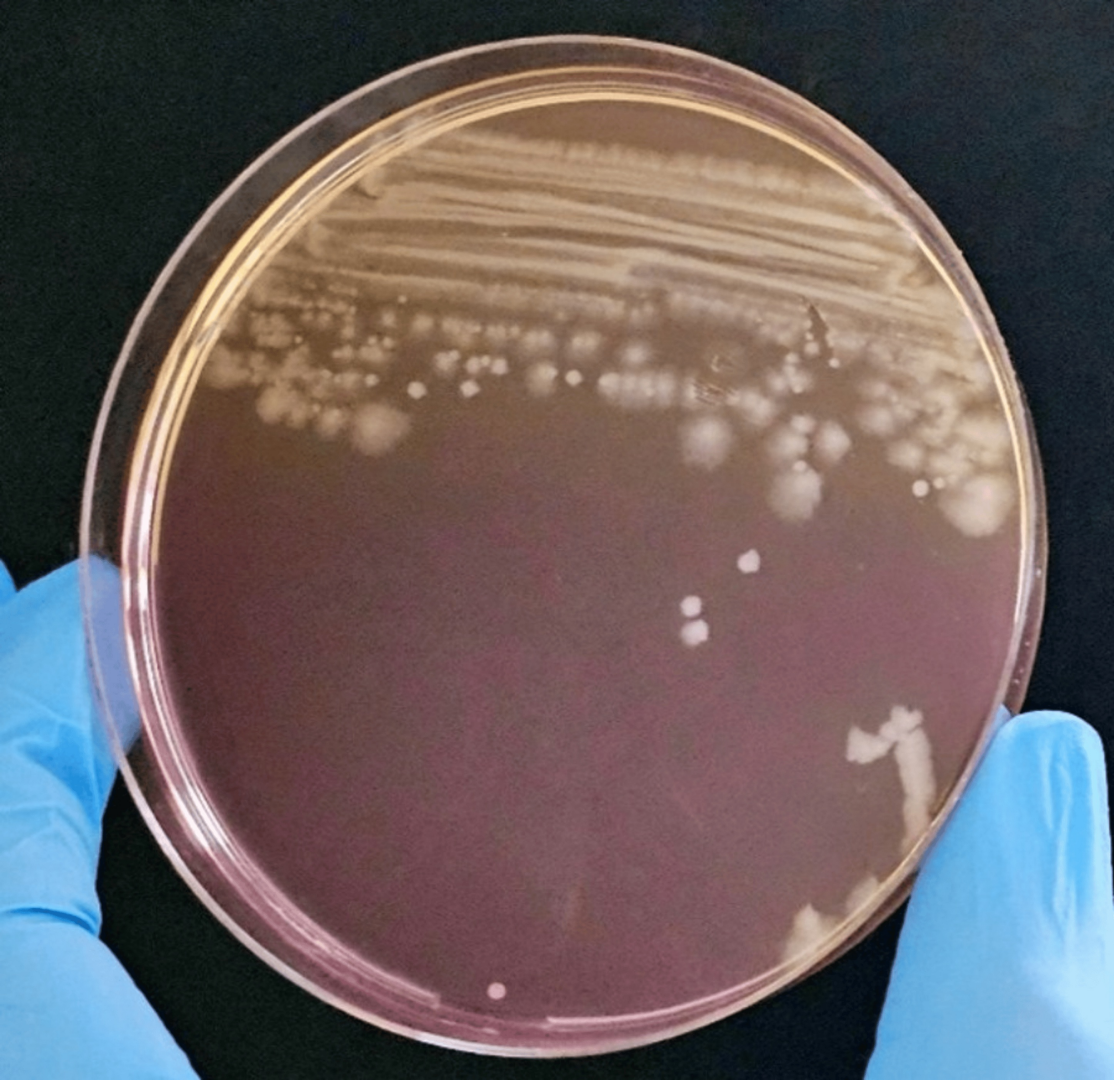
Blood culture Non-lactose fermented colony (round and feathery) in MacConkey agar.

Blood and stool samples from the patient were analyzed at the microbiology laboratory. Blood cultures (aerobic and anaerobic) using BD BACTEC™ systems (Becton, Dickinson and Company, New Jersey, USA) revealed gram-negative bacilli after 10 hours and 19 minutes of incubation. Initial identification with matrix-assisted laser desorption ionization-time of flight (MALDI-TOF) suggested *Escherichia coli*; however, differentiation was unreliable due to the genetic similarity between *Shigella *and *Escherichia coli*. Subcultures on various media showed non-lactose fermenting colonies on MacConkey agar after 24 hours (Figure 1).

**Figure 2:**
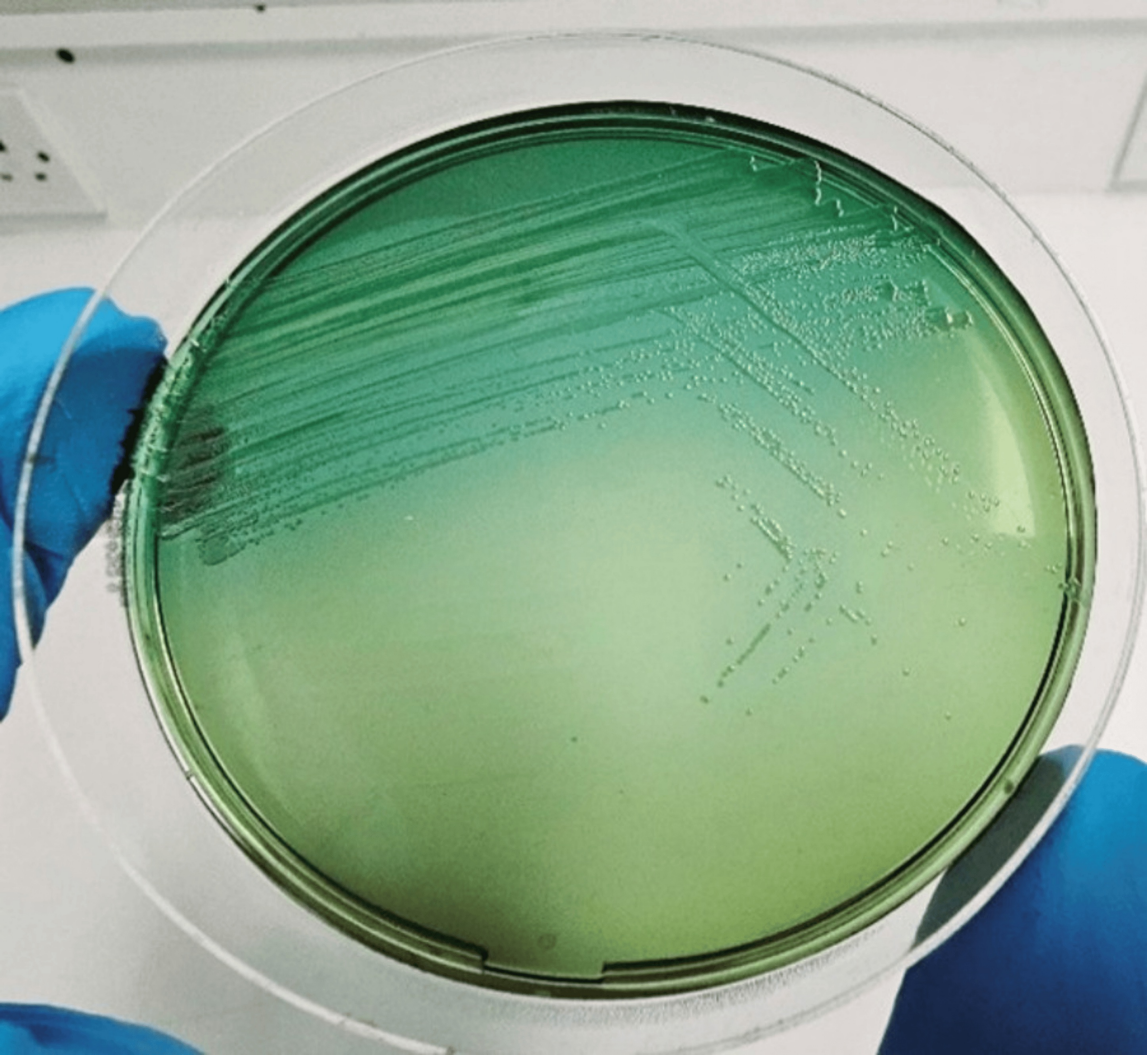
Stool culture Green colony in Hektoen agar.

Two distinct colony morphologies were identified as *Shigella sonnei* using the BD Phoenix™ M50 system (Becton, Dickinson and Company, New Jersey, USA). Susceptibility testing revealed ESBL production with resistance to cephalosporins, quinolones, and sulfamethoxazole-trimethoprim, while carbapenems remained effective. Confirmation was achieved through *Shigella *serotyping, which identified the organism as *Shigella sonnei*. Stool culture findings were consistent with the blood culture, isolating *Shigella sonnei* on MacConkey and Hektoen agar. BioFire® FilmArray® GI Panel (bioMérieux, Marcy-l'Étoile, France) directly from the stool sample further confirmed the identification (Figure 2).

The empirical treatment with ciprofloxacin 400 mg intravenous (IV) twice daily was initially started. Later, based on sensitivity results, ciprofloxacin was discontinued, and the treatment was adjusted to ertapenem 1 g IV daily. The infectious disease team recommended a 10-day course of ertapenem. Follow-up blood cultures 72 hours after initiating ertapenem confirmed clearance of the infection. The patient showed clinical improvement by day two and was completely asymptomatic by day three. He was discharged in good condition after 10 days, with a scheduled follow-up appointment.

## Discussion

*Shigella *species are gram-negative, nonmotile, facultatively anaerobic rods classified under the family *Enterobacteriaceae*, with four primary species: *Shigella dysenteriae* (serogroup A), *Shigella flexneri* (serogroup B), *Shigella boydii* (serogroup C), and *Shigella sonnei* (serogroup D) [1]. *Shigella sonnei* can be uniquely identified by its ornithine decarboxylase production, which helps distinguish it from other species [1]. The severity of illness caused by *Shigella *varies depending on the species and is associated with toxin production [2]. Notable toxins include ShET2 (found in all species), ShET1 (produced by *Shigella flexneri* 2a), and Shiga toxin (mainly produced by *Shigella dysenteriae* *1*, but occasionally by *Shigella flexneri* and *Shigella sonnei*) [10,11]. These toxins disrupt intestinal function by promoting fluid and electrolyte secretion, although severe disease can also occur in strains that do not produce toxins [11].

Due to its acid resistance,* Shigella *is primarily transmitted via the fecal-oral route, with a low infectious dose of 10-100 organisms. Transmission occurs through close contact, sexual activity (especially among men who have sex with men), and ingestion of contaminated food or water [4,12]. The incubation period is typically one to three days, with symptoms including fever, abdominal pain, and diarrhea, which may progress from watery to bloody with mucus [13,14]. Severity varies, with *Shigella sonnei* typically causing mild watery diarrhea, while *Shigella flexneri* and *Shigella dysenteriae* often lead to more severe dysenteric symptoms [14]. In immunocompetent individuals, the disease is usually self-limited and lasts up to seven days [15].

*Shigella *bacteremia is rare, with an incidence of 0% to 7%, occurring more frequently in vulnerable populations such as young children, the elderly, and individuals with comorbidities like diabetes [5,6]. In a study of 22 adult cases, one-third of patients were over 65 years old, and more than half had underlying conditions [16]. Interestingly, while HIV infection does not significantly increase the risk of *Shigella *bacteremia, it is associated with higher mortality in malnourished children [7].

Diagnosis of *Shigella *infection is often based on clinical symptoms, including diarrhea, abdominal pain, and fever, along with relevant epidemiologic exposure [17]. Stool examination may reveal white and red blood cells, suggesting infection. Stool culture remains the gold standard for diagnosis, as it allows bacterial isolation and antimicrobial susceptibility testing [17]. Polymerase chain reaction (PCR) testing is increasingly used but does not provide susceptibility data, so culture remains essential for guiding treatment [18,19]. Given the rise in drug resistance, antimicrobial susceptibility testing is crucial for tailoring therapy.

*Shigella *has developed resistance to several antibiotics, including fluoroquinolones, cephalosporins, and azithromycin, mainly through plasmid-mediated gene transfer. Resistance is more common in high-risk groups, including men who have sex with men, individuals with HIV, the homeless, and travelers to regions with high rates of infection [8,20]. Antibiotic-resistant *Shigella *strains are a significant concern globally, especially in Asia, Africa, and South America [12,21,22]. A study by the Georgia Department of Public Health from 2002 to 2012 found that only 0.64% of *Shigella* cases resulted in bacteremia, with a significant proportion of affected individuals being HIV-positive [23]. Our case is unique because the patient was immunocompetent, HIV-negative, and had no underlying conditions, which distinguishes him from the typical at-risk population.

The selection of antibiotics for treating *Shigella *infections depends on the patient's risk of resistance, which can be influenced by demographics, local resistance patterns, and exposure history [24]. Fluoroquinolones are recommended in cases without apparent risk factors, while carbapenems are preferred for severe infections or immunocompromised patients. 

## Conclusions

This case reports an instance of *Shigella *bacteremia with ESBL production in an immunocompetent adult in Qatar. It highlights the threat of antibiotic-resistant *Shigella*, particularly fluoroquinolones and cephalosporins. The case underscores the importance of timely diagnosis, susceptibility testing, targeted treatment, and preventive measures like proper hygiene and water sanitation. Research and surveillance are crucial to addressing resistance patterns in Qatar and similar regions.
